# Effects of an Innovative Telerehabilitation Intervention for People With Parkinson's Disease on Quality of Life, Motor, and Non-motor Abilities

**DOI:** 10.3389/fneur.2020.00846

**Published:** 2020-08-13

**Authors:** Sara Isernia, Sonia Di Tella, Chiara Pagliari, Johanna Jonsdottir, Carlotta Castiglioni, Patrizia Gindri, Marco Salza, Cristina Gramigna, Giovanna Palumbo, Franco Molteni, Francesca Baglio

**Affiliations:** ^1^IRCCS Fondazione don Carlo Gnocchi ONLUS, Milan, Italy; ^2^Fondazione Opera San Camillo Presidio Sanitario San Camillo, Turin, Italy; ^3^Villa Beretta Rehabilitation Center, Costa Masnaga, Italy

**Keywords:** rehabilitation, technology, telerehabilitation, nervous system disease, Parkinson's disease, digital health, continuity of care, quality of life

## Abstract

Parkinson's disease (PD) often leads to multifactorial motor and non-motor disabilities with resultant social restrictions. Continuity of care in this pathology, including a tailored home rehabilitation, is crucial to improve or maintain the quality of life for patients. The aim of this multicenter study was to test in a pilot sample of PD patients the efficiency and efficacy of the Human Empowerment Aging and Disability (HEAD) program. The virtual reality HEAD program was administered in two consecutive phases: (1) in clinic (ClinicHEAD, 12 45-minutes sessions, 3 sessions/week); (2) at home (HomeHEAD, 60 45-minutes sessions, 5 sessions/week). Thirty-one PD outpatients were enrolled [mean age (SD) = 66.84 (9.13)]. All patients performed ClinicHEAD, and after allocation (ratio 1:2) were assigned to the HomeHEAD or the Usual Care (UC) group. Motor, cognitive and behavioral outcome measures were assessed at enrollment (T0), at hospital discharge (T1), at 4 (T2) and 7 (T3) months after baseline. After ClinicHEAD (T1 vs. T0 comparison) a significant (*p* < 0.05) improvement in functional mobility, balance, upper limb mobility, global cognitive function, memory, quality of life and psychological well-being was observed. After the HomeHEAD intervention there was an additional enhancement for upper limb mobility. At T3 follow-up, the UC group that did not continue the HEAD program at home showed a worsening with respect to the HomeHEAD group in balance and functional mobility. Furthermore, in the HomeHEAD group, a positive association was observed between adherence, mental and physical health (SF-12). A trend was also registered between adherence and positive affect. The digital health patient-tailored rehabilitation program resulted in improving motor and non-motor abilities and quality of life in clinical setting, enhancing the motor function in telerehabilitation at home, and maintaining the non-motor abilities and quality of life at follow-up. In the near future, people with PD can be supported also at home with individualized rehabilitation strategies for a better quality of life and wellbeing along with lower costs for society.

## Introduction

Parkinson's disease (PD) is a progressive neurodegenerative condition, causing primarily an impairment in the motor system (1). It is the second most common neurodegenerative disorder after Alzheimer's disease and affects approximately seven million people globally (2). Moderate to severe dopaminergic neuronal loss that affects the *substantia nigra pars compacta* area may be considered the principal cause of the motor clinical manifestations, such as bradykinesia plus rigidity and resting tremor from the early stages of the disease (3). Even though PD is still considered a paradigmatic movement disorder, it is accompanied by remarkable non-motor symptoms, such as cognitive impairment, behavioral disturbances, hyposmia, sleep disorders, and autonomic dysfunction, even from the early stages of the pathology (4–6). Non-motor symptoms may become dominant with the progression of the disease in the clinical manifestation, with significant implications on quality of life and caregiver burden (7).

Among non-motor symptoms, cognitive impairment, which develops in dementia in up to 80 % of patients in the long term (8–10), can be characterized by a dysfunction in different domains, covering executive functions, working memory, attention, visuospatial abilities, and language (11). In particular, executive functions are essential for goal-directed activities (12), and executive dysfunction in PD, principally ascribed to damage of the dorsal striatum and putamen, and resulting in a functional alteration of dorsolateral fronto-subcortical circuits (13), may affect a great variety of goal-directed behaviors. As a result, patients may encounter difficulties with planning, organizational skills, and concentration while undertaking daily activities. Furthermore, the impairment in visuospatial abilities is related to deficits in other cognitive domains (for example, executive functions and verbal memory), postural control and gait, along with functional disability in non-demented patients with mild to moderate PD (14).

Considering the broad spectrum of motor and non-motor symptoms, the management of people with PD needs multidisciplinary interventions in order to provide patients with independent functioning as long as possible. Also, engaging in physical and cognitive exercise for the long term is of utmost importance to mitigate the course of the pathology and to prevent the need for PD medications at the early stages (15). At the same time, long-lasting health care is extremely expensive and often patients are not able to bear the associated costs (16). Recently, a randomized controlled study shed light on the beneficial effect of rehabilitation interventions in a real world setting on clinical deficits in PD (17).

To answer the need of implementing health interventions in the continuity of care together with decreasing health care costs for the chronic management of PD (18, 19), digital health offers several potential advantages. Accordingly, recent contributions described the growing implementation and diffusion of digital health solutions (20), suggesting an imminent integration of this digital revolution into the health care system (21). Especially, three main directions are being adopted: to guarantee a higher accessibility to health care services through telehealth to slow down related costs; to expand the target of intervention mainly focused on acute conditions to also chronic pathologies; to move the setting of rehabilitation from inside the clinic to patient's home (22). This is in line with the recent plan of Sustainable Development Goals that called for an imminent consolidation of the healthcare system with digital technology (23). Moreover, the implication of digital health allows to act and promote lifestyle changes, by reaching patients in their everyday life setting (24). Concerning telerehabilitation interventions, the central role of a digital health platform is recognized, constituting the hub of clinic-home communication and allowing assessment, monitoring and feedback during the rehabilitation period (25, 26).

Recent work regarding the implementation and validation of digital health interventions for PD provide evidence for their beneficial effect on outcome measures and health care costs (17, 27). Furthermore, the perception of patients with PD regarding telemedicine is positive (28) indicating many strengths, such as the cost-related and time-dependence convenience and the possibility of telecommunication with clinicians (29). However, the refinement of digital health solutions with the goal to offer a patient-tailored intervention remains an on-going process (30). Moreover, the study of O'Connor et al. (31) created the digital health engagement model aiming at highlighting the key aspects to be considered to provide digital health products able to be endorsed and accredited by the clinical system.

Recently, a new multidimensional telerehabilitation protocol for chronic neurological disease has been implemented for the continuity of care, named the Human Empowerment Aging and Disability (HEAD) program. This digital health solution proposes a rehabilitation program in a virtual reality (VR) setting to enhance motor and cognitive abilities and quality of life. HEAD has already been shown to promote high adherence coupled with good usability of its technological system (32). However, studies investigating its effectiveness on treating PD-related clinical impairments are still lacking.

The aim of this study was to test the clinical effectiveness of the HEAD telerehabilitation protocol in patients with PD. First, we investigated the efficiency of the HEAD system, in terms of adherence and usability; second, we explored the impact of HEAD program on the outcome measures, such as motor, cognitive functions and quality of life.

## Methods

### Intervention Design

**Figure 1:**
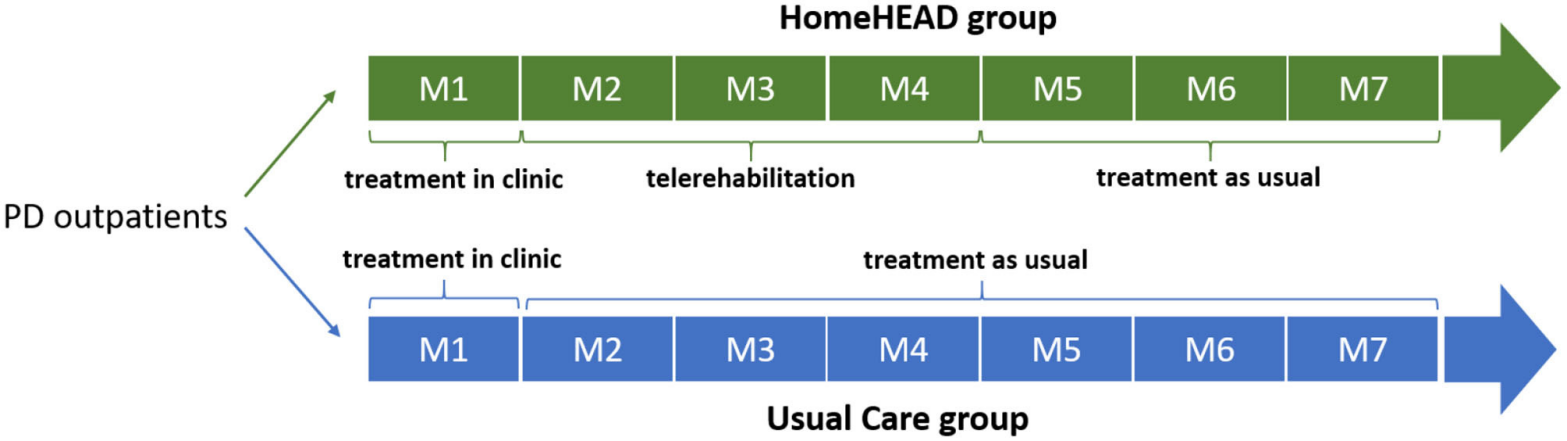
Timeline of the HEAD trial.

The study design was previously described in a recent work (32) and registered (ID: NCT03025126). Briefly, outpatients were involved in 1-month HEAD rehabilitation in the clinic, 45-min-session/3 times per week, for a total of 12 sessions (ClinicHEAD). Then, they were consecutively allocated to the HEAD telerehabilitation (HomeHEAD) or usual care condition (UC) with a ratio of 1:2 (this allocation procedure was due the limited availability of the technological kits). HomeHEAD consisted of a 3-month HEAD telerehabilitation, 45-min-session/5 times per week, in total 60 sessions. In the UC condition people performed physical activities they would usually do (Figure 1). Participants were assessed for efficiency and effectiveness measures at baseline (T0), after 1-month of ClinicHEAD (T1), after 3-months of HomeHEAD/UC (T2), and after 7 months from the enrollment (T3). The assessors were blind to patients' allocation and were unable to distinguish whether subjects received HomeHEAD treatment or treatment as usual (UC).

The study was approved by the local ethics committees of the three centers in which participants were recruited: the inter-company of the province of Lecco, Como and Sondrio, the Ethics Committee of IRCCS Don Gnocchi Foundation and the inter-company “Città della Salute e della Scienza” of Turin.

### The Treatment: HEAD Program vs. Usual Care

The HEAD program is a multidimensional rehabilitation for the enhancement of motor and cognitive functions of people with chronic neurological diseases, such as PD, Multiple Sclerosis and stroke [see for details on the HEAD protocol (32)]. Briefly, each rehabilitative session includes both motor and cognitive tasks, leisure and dual-task activities. These activities are patient-tailored and are conceived to improve balance, endurance, speed, and strength of both upper and lower limbs, executive functions, memory, language, and dual-task capabilities. The activities are embedded in short video clips to motivate the patients to carry out the rehabilitation. The video-clips constitute a reward, a short break or the material of the activity (for example to be memorized). Gaming technological devices are provided to perform activities in a VR scenario using Kinect (Microsoft, WA, USA) and Leap Motion (Leap Motion Inc., CA, USA) devices. Patients access the HEAD portal via Internet in order to perform rehabilitation sessions managed by clinicians in the HEAD digital health platform [for more details see (32)]. During ClinicHEAD, patients familiarized themselves with the HEAD technological kit in clinic and carried out the activities under the supervision of clinical professionals. After 1-month of ClinicHEAD, patients performed rehabilitation activities in the continuity of care at home (HomeHEAD). Technical issues and motivation were managed through periodic phone calls and the availability of the HEAD Help Desk.

Patients who were not allocated to the HomeHEAD were instructed to not take part in motor or cognitive activities related to rehabilitation different from what they usually do (Usual Care condition—UC). They were invited to follow health recommendations of the neurologists for their clinical conditions.

### Participants

Thirty-one patients were recruited in three clinics in North Italy: Valduce Hospital Villa Beretta Rehabilitation Center in Lecco, IRCCS Don Carlo Gnocchi Foundation in Milan and District Clinic San Camillo in Turin. In each clinical center, patients were enrolled during their periodical clinical visit by the neurologists. The inclusion criteria for the eligibility for participation in the study were: age <80 years, diagnosis of PD, stable pharmacological treatment for at least the past 3 months, Hoehn and Yahr (33) score ≤ 2. Exclusion criteria included a Mini-Mental State Examination score <20 (34), disabling pain, epilepsy, severe visual acuity and auditory perception, communication deficit, severe dysmetry and severe upper limb difficulties in passive range of motion. Before taking part in the study, patients read the information sheet of the study and gave their written informed consent.

In total, 31 people with PD were included in the study. All participants underwent 1 month of ClinicHEAD rehabilitation. Then, 11 patients were allocated to HEAD telerehabilitation while 20 people with PD were included in the UC condition. Three patients in the UC group were not evaluated at T3 (see Supplementary Material: CONSORT Flow Diagram for details).

### Measurement

The assessment was performed to evaluate output and outcome measures to test *efficiency* and *effectiveness* of HEAD treatment, respectively.

#### Output Measures

To test *efficiency*, adherence to treatment was registered during ClinicHEAD and HomeHEAD through the number of sessions performed by participants. This datum was collected in the HEAD platform and allowed clinicians to monitor whether or not patients performed the telerehabilitation activities at home. In fact, information related to each patient's log in the HEAD session and his performance of scheduled rehabilitation activities was saved in the HEAD platform server (32). The 80% of sessions completed has been considered as the cut-off of a high adherence to treatment in the PD sample both for clinic (total sessions > 9) and home (total sessions ≥ 48) program. Also, perceived usability related to the HEAD technological kit was investigated through the System Usability Scale [SUS; (35)]. This scale measures the usability of technology systems and devices by administering 10 items with a 5-point Likert scale, for a total score ranging from 10 to 100. A guideline cut-off of 68 is reported as a good level of usability for the technological system.

#### Outcome Measures

The *effectiveness* assessment protocol comprised a multi-domain evaluation by measuring cognitive functions, motor abilities and quality of life. Primary outcomes were change in one measure for each domain assessed, as described below. Outcomes on all other scales and tests were secondary.

##### Motor functions assessment

Motor abilities were evaluated by a physiotherapist blind to the group's allocation of the patients with the following measures:

- Berg Balance Scale [BBS; (36)]. A test for the assessment of patient's static balance and his risk of falling through a 14-item 4-points scale, with a total score ranging from 0 to 56;
- Ten Meter Walk Test [10MWT; (37)]. A test for a quantitative analysis of the walking speed. The speed in meters *per sec* for a walk of 10 meters is measured. It is considered an assessment of functional mobility.
- Two Minute Walk Test [2MWT; (38)]. A test for a quantitative analysis of gait speed and endurance. The distance walked in 2 min is registered, as a functional mobility measure.
- Box and Block Test [BBT; (39)]. A test for the assessment of upper extremity function related to the activities of daily living. Individuals move as many blocks as possible from one compartment to another in 60 s. A score is obtained by counting the number of blocks moved during the 1-min interval.

2MWT score consisted of the primary outcome of the motor domain.

##### Non-motor functions assessment

The evaluation of cognitive functions was performed by a neuropsychologist blind to the group's allocation of the patients and comprised the following neuropsychological battery:

- Montreal Cognitive Assessment [MoCA; (40)]. It is a sensitive tool for global cognitive level assessment, by screening different domains, such as executive function, memory, language, visual-spatial abilities, attention, calculation, abstraction, spatial and temporal orientation. The total score ranges from 0 to 30. In this study, Conti's (40) correction was adopted to correct scores for age and level of education of individuals;
- Rivermead Behavioral Memory Test-Third Edition [RBMT-3; (41, 42)]. An ecological battery for the assessment of memory abilities. This test evaluates memory through ten tasks: (1) First and Second Names, presentation and delayed memory of names and faces, (2) Belongings, prospective memory consisting of remembering to ask regarding personal belonging at the end of the evaluation session, (3) Appointments, prospective memory task in which subject has to remember to ask two questions when an alarm rings, (4) Picture Recognition, delayed picture recognition against distractors, (5) Story, immediate and delayed recognition of short stories, (6) Face Recognition, delayed recall of faces against distractors, (7) Route, immediate and delayed recall of a short route previously performed with the experimenter, (8) Messages, immediate and delayed remembering to pick up an envelope and a book in the right place of the route, (9) Orientation and Date, questions related to persons, places and timing, (10) Novel Task, immediate and delayed recall of the sequential procedure showed by the examiner to make a star with pieces inside a template. In addition to the sub-test scores, a global memory index score can be obtained.

MoCA score was considered the primary outcome of the cognitive domain.

##### Quality of life and psychological well-being assessment

The evaluation of quality of life of PD were performed with:

- Short Form Health Survey [SF-12; (43)]. This Scale measures a global assessment of the health-related quality of life from the patients' perspective. Consisting of 12 items, it assesses Mental Health and Physical Health Score.
- Positive Affect and Negative Affect Schedule [PANAS; (44)]. This is a schedule for the positive and negative affective states measure. This scale allows the measuring of the level of positive and negative affect. 20 5-points Likert scale items are administered, and 2 sub-scales are obtained: positive affect and negative affect, ranging 0–50 each.

The scores of the SF-12 Mental and Physical domains represented the primary outcome of the quality of life domain.

#### Statistical Analyses

All statistical analyses on output and outcome measures were performed using IBM SPSS Statistics software (Version 24). Descriptive statistics were employed to evaluate efficiency and effectiveness data. To evaluate adherence, we computed the percentage of subjects who reached at least the 80% of completed sessions. Multiple imputation by chained equations was performed to replace missing values in order to address potential biases due to incomplete follow-up. The multiple imputation procedure was applied in accordance with guidelines recommended for clinical trial data (45), which suggests that multiple imputation should not be used with a percentage of missing values more than 40%. In the imputation model were included all primary and secondary outcomes. Fifty datasets after imputation of plausible values to missing data were generated. Each primary/secondary outcome was considered and analyzed separately. We assessed patients' longitudinal performance at four time points: T0, T1, T2, and T3. Due to the multiple imputation procedure available in SPSS, we calculated change scores (Δvalues) from T1-T0, T2-T1, T3-T1, T3-T0 and after that we adopted paired and independent sample *t*-tests. Specifically, paired sample *t*-tests were performed to compare T1 vs. T0 outcome measures in the whole sample of PD patients, and T2 vs. T1 in the HomeHEAD group, while a two-sample *t*-test was performed to compare HomeHEAD and UC groups. Effect sizes were calculated for the primary outcomes. To evaluate the efficacy of HEAD treatment on quality of life and psychological well-being, we computed partial correlations. We explored the relationship between the adherence to the HEAD program and the Physical and Mental Health Scores of the SF-12 Health Survey at T1 in the whole group (ClinicHEAD) and at T2 and T3 separately in the UC and the HomeHEAD groups, controlling for the evaluation at the previous timepoint. An overall alpha-level of 0.05 was fixed for each statistical test. As suggested by Feise (46), regardless of *p*-value adjustments in testing that involves comparing treatments using multiple outcome measures with univariate statistical method to reach a reasonable conclusion, we calculated the magnitude of effects and we included effect sizes in **Tables 2**–**4**. Effects sizes (*Cohen's d*) were interpreted as follows: 0.2 to 0.49 as a small effect; 0.5 to 0.79 as an intermediate effect; 0.8 and higher as a strong effect (47).

## Results

### Participants

Baseline demographical and clinical characteristic of our sample is reported in Table 1. The UC and HomeHEAD groups did not differ for age, years of education and sex (all *p*-values > 0.05).

**Table 1:** Sociodemographic characteristics of the whole PD sample (ClinicHEAD group), and UC and HomeHEAD groups.

	**ClinicHEAD**	**UC**	**HomeHEAD**	**UC vs. HomeHEAD *p***
*N*	31	20	11	
Age [Mean (SD)]	66.84 (9.13)	67.55 (9.33)	65.55 (9.06)	0.563
Education [Mean (SD)]	11.77 (4.33)	12.05 (4.22)	11.27 (4.69)	0.637
Sex (M/F, %)	17/14 (54.8%, 45.2%)	13/7 (65.0%, 35%)	4/7 (36.4%, 63.6%)	0.125
**MOTOR FUNCTIONING**				
2MWT	131.23(36.72)	131.00 (36.47)	131.64 (38.94)	0.965
BBS [Mean (SD)]	48.67 (6.45)	48.37 (6.80)	49.18 (6.06)	0.733
BBT—dominant [Mean (SD)]	41.48 (13.56)	39.75 (14.88)	44.64 (10.66)	0.338
BBT—non dominant [Mean (SD)]	41.74 (13.59)	41.15 (15.32)	42.82 (10.32)	0.747
10MWT [Mean (SD)]	7.02 (4.90)	6.52 (2.43)	7.86 (7.60)	0.475
**NON-MOTOR FUNCTIONING**				
MoCA [Mean (SD)]	21.94 (2.82)	22.27 (2.64)	21.35 (3.16)	0.386
RBMT-GMI [Mean (SD)]	83.94 (17.81)	82.25 (16.42)	87.00 (20.58)	0.481

*UC, usual care; BBT, Box and Block Test; 2MWT, 2-Meter Walk Test; BBS, Berg Balance Scale; MoCA, Montreal Cognitive Assessment; RBMT-GMI, Rivermead Behavioral Memory Test-Third Edition—Global Memory Index; M, Mean; SD, Standard Deviation; p, p-value*.

### Output Measures

#### Adherence

Twenty-six subjects (83.9%) demonstrated a high adherence to ClinicHEAD in terms of a rate of completed sessions above 80%. Moreover, 72.7% of HomeHEAD's participants (8 subjects vs. 11) reached the cut-off score of adherence.

#### Usability

Data showed a usability score over cut-off after both ClinicHEAD and HomeHEAD treatments. Results from the SUS showed a median value of 70.00 (25–75th percentile 60.00–82.50) at T1, and 85.00 (25–75th percentile 77.50–92.50) at T2.

### Outcome Measures

#### Changes in Motor and Non-motor Outcomes After ClinicHEAD Program (T1 vs. T0)

The T1 vs. T0 comparison showed a significant improvement in functional mobility (2MWT: *t* = 2.254; *df* = 30; *p* = 0.024; *Cohen's d* = 0.41); balance (BBS: *t* = 2.059; *df* = 30; *p* = 0.043; *Cohen's d* = 0.37); upper limb mobility (BBT – dominant: *t* = 4.680; *df* = 30; *p* < 0.001; *Cohen's d* = 0.84; and non-dominant: *t* = 2.836; *df* = 30; *p* = 0.005; *Cohen's d* = 0.51); global cognitive function (MoCA: *t* = 2.139; *df* = 30; *p* = 0.032; *Cohen's d* = 0.38); memory (RBMT: *t* = 3.645; *df* = 30; *p* < 0.001; *Cohen's d* = 0.66). Table 2 summarizes the results.

**Table 2:** Effectiveness of ClinicHEAD program (T0 vs T1).

	**T0**		**T1**		***p***	***Cohen's d***
	**Mean**	**SD**	**Mean**	**SD**		
**PRIMARY OUTCOME**						
**Motor**						
2MWT	131.23	36.72	140.30	37.54	**0.024**	0.41
**Non-motor**						
MoCA	21.94	2.82	22.88	3.51	**0.032**	0.38
**SECONDARY OUTCOME**						
**Motor**						
BBS	48.67	6.45	50.43	6.00	**0.040**	0.37
BBT—dominant	41.48	13.56	46.39	13.73	**<0.001**	0.84
BBT—non dominant	41.74	13.59	44.81	13.74	**0.005**	0.51
10MWT	7.02	4.90	5.96	2.12	0.156	0.26
**Non-motor**						
RBMT-GMI	84.48	18.29	92.10	17.46	**<0.001**	0.66

*p-values < 0.05 are reported in bold. BBT, Box and Block Test; 2MWT, 2-Meter Walk Test; BBS, Berg Balance Scale; MoCA, Montreal Cognitive Assessment; RBMT-GMI, Rivermead Behavioral Memory Test-Third Edition—Global Memory Index; SD, Standard Deviation; p, p-value*.

#### Changes in Motor and Non-motor Outcomes After HomeHEAD Program (T2 vs. T1)

In the HomeHEAD group (*N* = 11), the T2 vs. T1 comparison showed an additional enhancement for the upper limb mobility (BBT – non-dominant: *t* = 2.861; *df* = 10; *p* = 0.004; *Cohen's d* = 0.86). The positive effects obtained after ClinicHEAD program were also maintained in all other outcome measures in the HomeHEAD group (Table 3).

**Table 3:** Effectiveness of HomeHEAD program (T1 vs T2).

	**T1**		**T2**		***p***	***Cohen's d***
	**Mean**	**SD**	**Mean**	**SD**		
**PRIMARY OUTCOME**						
**Motor**						
2MWT	139.27	29.19	140.91	41.88	0.744	0.10
**Non-motor**						
MoCA	22.37	4.98	23.47	3.15	0.346	0.28
**SECONDARY OUTCOME**						
**Motor**						
BBS	51.64	4.82	50.73	5.78	0.198	0.39
BBT—dominant	48.36	12.11	49.55	9.74	0.472	0.22
BBT—non dominant	44.82	11.29	49.27	10.52	**0.004**	0.86
10MWT	5.46	1.10	5.71	1.83	0.407	0.25
**Non-motor**						
RBMT-GMI	90.30	19.32	90.10	19.13	0.708	0.11

*p-values < 0.05 are reported in bold. UC, usual care; BBT, Box and Block Test; 2MWT, 2-Meter Walk Test; BBS, Berg Balance Scale; MoCA, Montreal Cognitive Assessment; RBMT-GMI, Rivermead Behavioral Memory Test-Third Edition—Global Memory Index; SD, Standard Deviation; p, p-value*.

#### Changes in Motor and Non-motor Outcomes: Comparison Between UC and HomeHEAD Group

After ClinicHEAD treatment (ΔT1-T0) the UC group did not differ from the HomeHEAD group (Table 4). After home program (ΔT2-T1) differences between the HomeHEAD group and the UC were observed in upper limb mobility (BBT – non-dominant: *t* = −3.169; *df* = 29; *p* = 0.002; Cohen's *d* = 1.19) and functional mobility (2MWT: *t* = −2.130; *df* = 29; *p* = 0.033; *Cohen's d* = 0.80). Also, a trend of effect on dominant hand dexterity was observed after HomeHEAD (BBT – dominant: *t* = −1.730; *df* = 29; *p* = 0.084; Cohen's *d* = 0.65).

**Table 4:** Comparison between UC and HomeHEAD groups on neuropsychological and motor measures after ClinicHEAD program (ΔT1-T0), after 3-months of HomeHEAD/ UC (ΔT2-T0), after 6-months from ClinicHEAD (ΔT3-T1), and after 7 months from the enrolment (ΔT3-T0) using independent sample *t*-test.

	**ΔT1-T0**					**ΔT2-T1**					**ΔT3-T1**					**ΔT3-T0**				
	**UC**		**HomeHEAD**			**UC**		**HomeHEAD**			**UC**		**HomeHEAD**			**UC**		**HomeHEAD**		
	**Mean**	**SD**	**Mean**	**SD**	***p* (*Cohen's d*)**	**Mean**	**SD**	**Mean**	**SD**	***p* (*Cohen's d*)**	**Mean**	**SD**	**Mean**	**SD**	***p* (*Cohen's d*)**	**Mean**	**SD**	**Mean**	**SD**	***p* (*Cohen's d*)**
**PRIMARY OUTCOME**																				
**Motor**																				
2MWT	9.89	23.97	7.64	21.01	0.794 (0.10)	−10.71	14.19	1.64	16.60	**0.033** (0.80)	−20.71	36.99	−0.91	17.09	**0.045** (0.75)	−12.35	49.99	6.73	25.05	0.163 (0.54)
**Non-motor**																				
MoCA	0.75	1.80	1.27	3.38	0.572 (0.21)	−0.50	2.00	1.10	2.88	0.117 (0.61)	−0.10	3.04	0.09	3.45	0.867 (0.06)	0.90	3.26	1.36	2.34	0.683 (0.15)
**SECONDARY OUTCOME**																				
**Motor**																				
BBS	1.37	5.52	2.45	3.56	0.554 (0.22)	−5.00	9.64	−0.91	2.34	0.158 (0.65)	−7.17	14.09	−0.73	2.28	**0.045** (0.75)	−5.72	13.78	1.73	3.80	**0.023** (0.85)
BBT—dominant	5.55	5.94	3.73	5.71	0.408 (0.32)	−2.35	5.17	1.18	5.46	0.084 (0.67)	−1.22	9.08	1.36	4.52	0.307 (0.39)	4.00	9.54	5.09	6.99	0.744 (0.12)
BBT—non dominant	3.65	5.97	2.00	6.24	0.469 (0.28)	−1.65	4.76	4.45	5.16	**0.002** (1.19)	0.33	6.75	2.45	5.66	0.380 (0.33)	3.89	8.24	4.45	8.81	0.863 (0.07)
10MWT	−0.28	1.08	−2.41	6.75	0.173 (0.52)	0.19	0.78	0.25	1.00	0.899 (0.05)	1.31	3.64	0.43	1.40	0.355 (0.35)	1.14	3.96	−1.98	5.49	0.079 (0.68)
**Non-motor**																				
RBMT-GMI	9.67	13.16	4.27	11.49	0.247 (0.44)	1.33	16.38	−0.20	10.26	0.794 (0.10)	5.25	11.93	1.73	8.60	0.387 (0.33)	10.39	17.94	6.00	11.36	0.459 (0.28)

*p-values < 0.05 are reported in bold. UC, usual care; BBT, Box and Block Test; 2MWT, 2-Meter Walk Test; BBS, Berg Balance Scale; MoCA, Montreal Cognitive Assessment; RBMT-GMI, Rivermead Behavioral Memory Test-Third Edition—Global Memory Index; SD, Standard Deviation; p, p-value*.

At the follow-up, the UC showed a worsening compared to the HomeHEAD group in balance (BBS, ΔT3-T1: *t* = −2.006; *df* = 29; *p* = 0.045; *Cohen's d* = 0.75; ΔT3-T0: *t* = −2.273; *df* = 29; *p* = 0.023; *Cohen's d* = 0.85) and functional mobility (2MWT, ΔT3-T1: *t* = −2.007; *df* = 29; *p* = 0.045; *Cohen's d* = 0.75). Table 4 summarizes the results.

#### Quality of Life and Psychological Well-Being

The T1 vs. T0 comparison showed a significant improvement in the Mental Health Score of the SF-12 Health Survey (*t* = 2.181, df = 29; *p* = 0.029; *df* = 30; *p* = 0.019; *Cohen's d* = 0.39) and mood (PANAS positive affect: *t* = 2.349; *df* = 30; *p* = 0.019; *Cohen's d* = 0.42).

Investigating the relationship between SF-12 scores and adherence at HEAD treatment we observed:

- ClinicHEAD group: no significant correlation after clinic treatment (T1);
- HomeHEAD group:
  ° a positive partial correlation between the percentage of completed sessions at home and the Mental Health Score of the SF-12 Health Survey at T2 including T1 Mental Health Score as covariate (*r* = 0.743; *p* = 0.022);
  ° a positive partial correlation between the total completed sessions (Clinic+Home sessions) and the Physical Health Score of the SF-12 Health Survey at T3 including T0 Mental Health Score as covariate (*r* = 0.790; *p* = 0.034).
- UC group: a negative partial correlation between the total completed sessions and the Mental Health Score of the SF-12 Health Survey at T3 including T0 Mental Health Score as covariate (*r* = −0.778; *p* < 0.001).

Moreover, investigating the relationship between PANAS scores and adherence at HEAD treatment we observed:

- ClinicHEAD group: a positive partial correlation was observed between adherence of ClinicHEAD sessions and positive affect at T1, including PANAS score at T0 as covariate (*r* = 0.417; *p* = 0.022).
- HomeHEAD group: a trend was registered between the total number of completed sessions and positive affect at T3, including PANAS score at T0 as covariate (*r* = 0.578; *p* = 0.080)

UC group: no significant correlation at T2 and T3.

## Discussion

Digital technology is allowing innovative ways of rehabilitation care for chronic neurological diseases (18, 19), such as PD. Beneficial effects of telerehabilitation have recently been described (17, 27, 29). Given the growing effort spent in implementing increasingly patient-tailored rehabilitation in digital health continuity of care (30, 31), evidence of its effectiveness is needed (48).

Technological usability represents a key prerequisite allowing for the enactment of rehabilitation at home (49). Notably, Palacholla et al. (50) included the lack of technology usability and technical support as a barrier to digital health adoption. Our study supported the technological suitability of the HEAD kit for telerehabilitation by the point of view of patients. Globally, our efficiency findings shed light on the suitability of the HEAD telerehabilitation for PD.

At the same time, high adherence (>80% of all sessions) to HEAD treatment in clinic and at home was reported. Given the active role of the patient in telerehabilitation, adherence is a critical issue, particularly in the home setting where the patient is involved in the management of his/her health care. Without adherence, the patient loses the choices to embrace the full range of benefits related to the continuity of care. In line with this, several studies have focused on factors enhancing positive effects of e-patient activities, named as the non-medical people involved in own healthcare management by technological systems (51, 52). Also, adherence to treatment, mirroring patient's motivation, reflects the patient's empowerment in his/her own health management, in line with the phenomenon of e-patients (53). Interestingly, our data demonstrated a direct relationship between changes in Mental and Physical Health Scores of the SF-12 Health Survey and adherence, in terms of a major amelioration of quality of life in patients who consistently adhered to the HEAD program at home. Whereas, a negative association was observed for the Mental Health Score of the SF-12 Health Survey in the UC group. This finding supports the direct effect of telerehabilitation with VR tools to provide a motivating environment, which promotes greater adherence to an intensive treatment over a long-term period (54).

With respect to the motor and non-motor outcome measures of the trial, our effectiveness results are favorable regarding the HEAD program maintenance and amelioration of PD-related deficits. Especially, the first step of HEAD rehabilitation program (ClinicHEAD) suggests positive influence on motor, non-motor and well-being domains considered. In fact, after 1 month of HEAD rehabilitation in the clinic, PD participants obtained positive results in terms of both upper and lower functional mobility, balance, global cognitive level, memory, positive affect, and mental health. The multidimensional enhancement reflects improvements across the wide spectrum of the PD-related symptoms, which typically start with difficulties involving mainly the motor sphere and subsequently progress to disabling non-motor manifestations (4–7). Thus, our results provide further support in favor of rehabilitation's benefits in PD, even in the initial mild-to-moderate phases of the disease (55).

Considering the second step of HEAD rehabilitation program at home (HomeHEAD), results revealed an additional improvement in motor functioning (functional mobility and the manual gross motor functionality) along with the maintenance of the motor and non-motor performance achieved after 1 month of ClinicHEAD. On the contrary, the UC group worsened in terms of functional mobility. These data confirm the evidence of a recent meta-analysis that found telehealth effects especially with respect to motor functions (55, 56). Notably, our results provide additional evidence that well-structured rehabilitation treatment at home is efficacious (57–59).

Accordingly, our HomeHEAD's participants seemed to maintain the functioning achieved even after 3-months from the end of the telerehabilitation, and especially in terms of equilibrium (Berg Balance Scale). This result fosters the potential of the HomeHEAD program in decreasing incidents of falling, one of the most frequent complications of the disease. Also, the direct association observed between psychological well-being/quality of life and the adherence to telerehabilitation treatment at home provided an additional explanation of the maintenance of the motor capability. Indeed, previous work has underlined a link between subjective well-being and motor impairment (60).

A key and innovative feature of HEAD rehabilitation was the multidimensional treatment approach, also implemented at home, and the inclusion of patient-tailored digital contents. In fact, HEAD program combined motor, cognitive, and occupational activities developed with VR tools and multimedia contents. Previous examples of home-based rehabilitation for PD focused on single target domains such as motor difficulties, while the non-motor domains were less frequently included in rehabilitation protocols (61, 62). On the contrary, recent studies suggested the adoption of an inter-professional approach to provide a successful management of the disease including also the treatment of non-motor symptoms of PD (27, 61, 63). Moreover, all digital contents of HomeHEAD were not designed with a fixed schedule but were tailored based on needs of the single patient. After an initial evaluation, the staff was able to change the composition of exercises (i.e., level, intensity, and multimedia contents) at set time points in response to training task performance. Altogether, these findings underlined and supported the role of the personalized digital medicine in PD population for the delivery of an efficacious multidimensional tele-rehabilitation able to enhance and maintain motor and non-motor functioning and allowing for the continuity of care at home as well as to implement an individually tailored treatment (64).

This study is not without limitations. First, we could not perform a randomized clinical trial given the pilot exploratory nature of the trial and the limited availability of the technological kits. Also, due to this constrain, our sample size is small and our result should be considered with caution. Future trial should expand our results with a wider sample size and performing a randomized controlled trial. Second, the first step of the trial, ClinicHEAD, was performed in all PD sample, preventing us from the possibility to infer efficacy conclusions in comparison to a control group.

In conclusion, our results reflect the positive influence of a multidimensional rehabilitation approach to be performed at home for patients with PD by underlining its effects on motor and non-motor functioning. In the near future, the digital e-health approach will support the introduction of individualized rehabilitation strategies for PD patients, for a better quality of life and well-being, and lower costs for society.

## Data Availability Statement

The raw data supporting the conclusions of this article will be made available by the authors, without undue reservation.

## Ethics Statement

The studies involving human participants were reviewed and approved by Don Gnocchi Foundation Ethics Committee. The patients/participants provided their written informed consent to participate in this study. This clinical trial has been registered on clinicaltrials.gov, under ID: NCT03025126.

## Author Contributions

FB, MS, and FM conceived the study. CP, CC, and CG recruited sample and recruited the clinical evaluation. JJ, PG, and GP collected data. SD, SI, and FB performed analysis and interpreted results. FB, SD, and SI wrote the first draft of manuscript. All authors reviewed and approved the final manuscript.

## Conflict of Interest

The authors declare that the research was conducted in the absence of any commercial or financial relationships that could be construed as a potential conflict of interest.
