# Supplementary material for: Effects of an Innovative Telerehabilitation Intervention for People With Parkinson's Disease on Quality of Life, Motor, and Non-motor Abilities
Source: Front Neurol. 2020 Aug 13;11:846. doi: 10.3389/fneur.2020.00846 (PMC7438538; doi:10.3389/fneur.2020.00846)
Supplement: Supplementary file 1 [file Table_1.DOC]

**
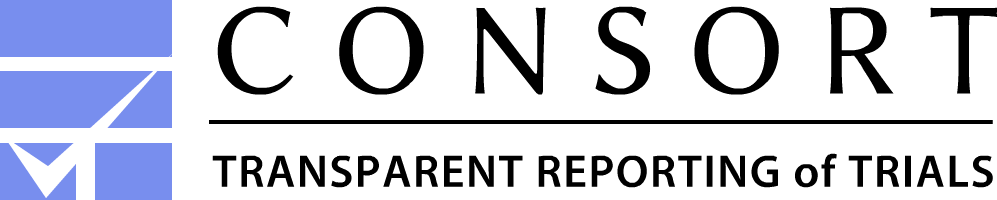
**

**CONSORT 2010 Flow Diagram**

**Allocation**

**Analysis**

**Follow-Up**

**Enrolment**

Excluded (n=0)

Analysed with multiple imputation (n=20)

Lost to follow-up (low compliance) (n=3)

♦ Unavailable at T2 (n=3)

Allocated to UC condition (n=20)

♦ Received allocated intervention (n=20)

♦ Did not receive allocated intervention (n=0)

Lost to follow-up (n=0)

Allocated to HEAD condition (n=11)

♦ Received allocated intervention (n=11)

♦ Did not receive allocated intervention (n=0)

Analysed with multiple imputation (n=11)

1:2 allocation (n=31)

Assessed for eligibility (n=31)
